# Polarized Macrophages and Their Exosomes: Implications for Autoimmune and Immune-Mediated Diseases

**DOI:** 10.3390/biology14101371

**Published:** 2025-10-08

**Authors:** Vincent G. Yuan

**Affiliations:** Department of Otolaryngology-Head and Neck Surgery, University of Pittsburgh Medical Center, Pittsburg, PA 15213, USA; vincentyuan@pitt.edu

**Keywords:** autoimmune diseases, exosomes, intercellular communication, macrophage polarization, inflammation, immunomodulation, rheumatoid arthritis, therapeutic delivery

## Abstract

**Simple Summary:**

Autoimmune diseases happen when the immune system attacks the body’s own tissues, causing chronic inflammation and damage. Macrophages, a type of immune cell, can either fuel inflammation or promote healing. They communicate with other cells through tiny packages called exosomes, which carry proteins and genetic signals. Exosomes from inflammatory macrophages can worsen tissue damage, while those from healing macrophages help calm inflammation and repair tissues. Research in conditions like rheumatoid arthritis, lupus, multiple sclerosis, inflammatory bowel disease, type 1 diabetes, and psoriasis shows these dual roles. Scientists are now exploring how to use macrophage exosomes as targeted therapies, either harnessing naturally healing exosomes or engineering them to deliver treatments. Understanding and applying this system could lead to safer, more precise, and personalized treatments, improving patient outcomes and reducing side effects from conventional drugs.

**Abstract:**

Autoimmune diseases result from dysregulated immune responses that mistakenly attack the body’s own tissues, causing chronic inflammation and progressive damage. Macrophages, with their remarkable plasticity, play key roles in both promoting and resolving inflammation, with pro-inflammatory M1 and anti-inflammatory M2 states shaping disease outcomes. Macrophage-derived exosomes have emerged as important mediators of intercellular communication, reflecting the functional state of their parent cells while influencing recipient cell behavior. Exosomes from M1 macrophages amplify inflammation through cytokines and microRNAs, whereas M2-derived exosomes support tissue repair and immune regulation. Studies in rheumatoid arthritis, lupus, multiple sclerosis, inflammatory bowel disease, type 1 diabetes, and psoriasis highlight their dual roles in pathology and resolution. In addition, macrophage exosomes can be engineered to deliver targeted therapeutic molecules, offering cell-free interventions with advantages in specificity, biocompatibility, and immunomodulation. This review summarizes current insights into macrophage-derived exosomes, their role in autoimmune pathogenesis, and emerging strategies to harness their therapeutic potential, highlighting their promise as precision-guided treatments for autoimmune diseases.

## 1. Introduction

Autoimmune diseases include a wide range of disorders marked by abnormal immune reactions that erroneously attack and harm the body’s own tissues [1,2,3]. Despite advances in immunology and therapeutic interventions, the etiology of many autoimmune conditions remains elusive, and current treatments often rely on generalized immunosuppression rather than precise immune modulation [4,5,6,7]. This gap highlights the need for a more comprehensive insight into immune system dynamics and the creation of more precise therapeutic approaches.

Among the myriad of immune cells implicated in autoimmunity, macrophages have garnered significant attention due to their phenotypic plasticity and pivotal roles in both initiating and resolving inflammation [8,9,10]. These versatile cells can assume different polarization states, typically categorized as pro-inflammatory (M1) or anti-inflammatory (M2), each displaying distinct functional characteristics [11]. M1 macrophages drive inflammation and tissue damage by releasing proinflammatory mediators such as TNF-α, IL-1β, and IL-6, whereas M2 macrophages are primarily involved in anti-inflammatory responses and the promotion of tissue repair [12,13]. The dynamic balance between these subsets profoundly influences the course of autoimmune diseases.

In recent years, extracellular vesicles, especially exosomes, have been recognized as key facilitators of communication between immune cells [14,15]. Originating from endosomal compartments and secreted by nearly all cell types, exosomes transport biologically active molecules such as proteins, lipids, and nucleic acids, which can significantly modulate the function of recipient cells [16,17].

Beyond macrophages, several other immune cells, including T cells, B cells, and dendritic cells, also release exosomes that play critical roles in shaping immune responses [18,19]. For example, T cell–derived exosomes can modulate antigen presentation and influence cytotoxic activity, B cell–derived exosomes can carry MHC peptide complexes to promote antibody responses, and dendritic cell–derived exosomes are known to enhance T cell priming and tolerance induction [20]. Collectively, these studies highlight that exosomes represent a shared communication mechanism across immune subsets.

What sets macrophage-derived exosomes (MφExos) apart, however, is their dual ability to both initiate and resolve inflammation depending on macrophage polarization status [21,22]. They are increasingly recognized not only as markers of macrophage activation states but also as functional mediators capable of modulating immune responses locally and systemically. The convergence of two research frontiers, macrophage polarization and exosome biology, has opened new avenues for understanding the pathophysiology of autoimmune diseases. Moreover, this intersection offers promising therapeutic opportunities, such as using exosome cargo to modulate immune responses or engineering exosomes as delivery vehicles for targeted interventions.

In this review, we explore the emerging role of polarized macrophage-derived exosomes in the context of autoimmune diseases. We begin with a brief overview of macrophage polarization and exosome biology and then examine how these vesicles contribute to the pathogenesis of autoimmunity. Finally, we discuss current and future therapeutic strategies that harness or target macrophage-derived exosomes to treat autoimmune disorders.

## 2. Macrophage Polarization and Functions

**Figure 1:**
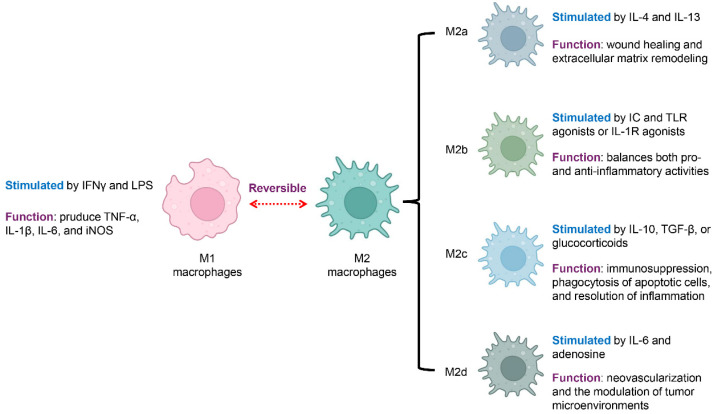
Macrophage Polarization States and Associated Functions. Macrophages exhibit distinct functional states that reflect their polarization. They can shift between a pro-inflammatory (M1) and an anti-inflammatory (M2) state, a process that is reversible and highly context-dependent. M1 macrophages are activated by IFN-γ and LPS and produce proinflammatory mediators, including TNF-α, IL-1β, IL-6, and iNOS. In contrast, M2 macrophages, or alternatively activated macrophages, are associated with anti-inflammatory functions and tissue repair. M2 macrophages are further classified into four subsets with specific functions: M2a macrophages, stimulated by IL-4 and IL-13, support wound healing and the remodeling of the extracellular matrix; M2b macrophages, activated by immune complexes along with TLR or IL-1R stimuli, regulate both pro- and anti-inflammatory responses; M2c macrophages, activated by IL-10, TGF-β, or corticosteroids, mediate immunosuppression, remove apoptotic cells, and support the resolution of inflammation, while M2d macrophages, driven by IL-6 and adenosine, enhance neovascularization and modulate the tumor microenvironment. Blue font indicates the stimulation condition, and violet font denotes the function.

Macrophages are innate immune cells central to host defense, tissue homeostasis, and the resolution of inflammation [23]. One of their most remarkable features is their ability to undergo polarization in response to environmental cues, acquiring distinct phenotypes and functions [24]. Rather than existing in fixed subsets, macrophage polarization represents a dynamic spectrum ranging from pro-inflammatory to anti-inflammatory states, underscoring their inherent plasticity (Figure 1) [25,26,27].

Classically activated macrophages, commonly called M1 macrophages, are stimulated by interferon-gamma (IFNγ) together with microbial components like lipopolysaccharide (LPS) [28,29]. These macrophages secrete abundant proinflammatory mediators, such as tumor necrosis factor-alpha (TNF-α), interleukin-1 beta (IL-1β), and IL-6, and express inducible nitric oxide synthase (iNOS) [30]. M1 macrophages play essential roles in microbial killing and antigen presentation. However, in autoimmune diseases, excessive M1 activation contributes to tissue damage and sustains chronic inflammation [9,26].

Alternatively activated macrophages, broadly referred to as M2 macrophages, are typically involved in anti-inflammatory functions, tissue repair, and the regulation of immune responses [30,31]. M2 polarization includes several subtypes, each triggered by distinct stimuli and exhibiting specific functions. M2a macrophages are stimulated by interleukin-4 (IL-4) and interleukin-13 (IL-13), supporting wound healing and extracellular matrix remodeling [32]. M2b macrophages develop in response to immune complexes (ICs) together with Toll-like receptor (TLR) activators, or via IL-1 receptor (IL-1R) stimulation, displaying a mixed cytokine profile that modulates both pro- and anti-inflammatory responses [33]. M2c macrophages, activated by IL-10, transforming growth factor-β (TGF-β), or corticosteroids, are linked to immune suppression, engulfment of dying cells, and the termination of inflammatory responses [31]. M2d macrophages, a subtype driven by IL-6 and adenosine, often referred to as tumor-supportive macrophages, are distinguished by their pro-angiogenic and cancer-fostering functions, aiding in new blood vessel formation and shaping the tumor microenvironment [34].

While the M1/M2 paradigm provides a useful conceptual model, it does not fully capture the functional diversity of macrophages in vivo (Table 1). Macrophages frequently adopt hybrid or transitional phenotypes and perform roles that extend beyond a simple pro- versus anti-inflammatory axis. Importantly, macrophage-derived exosomes reflect and amplify these diverse functions: they shape innate immune responses (for example by modulating dendritic cell maturation, NK cell activity, and neutrophil recruitment) [20,35,36], influence adaptive immunity (including B cell activation and antibody production, T helper polarization, and Treg induction) [37,38,39,40], and mediate direct intercellular cross-talk within inflamed microenvironments [22,41,42]. Recognizing these broader functions reframes exosomes not simply as inflammatory conveyors but as context-dependent information carriers that link innate sensing to adaptive responses and tissue remodeling.

## 3. Exosomes from Different Macrophage Subtypes

**Figure 2:**
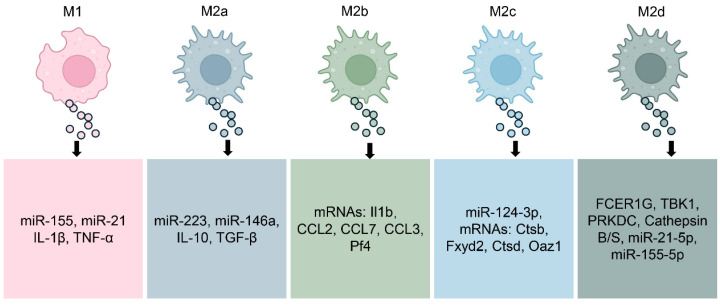
Molecular Composition of Exosomes by Macrophage Subtype. M1-polarized macrophages, well known for their pro-inflammatory activity, release exosomes enriched with mediators such as miR-155, miR-21, IL-1β, and TNF-α, which amplify inflammatory signaling. By contrast, exosomes derived from M2a macrophages, associated with tissue repair, carry molecules including miR-223, miR-146a, IL-10, and TGF-β. M2b macrophage–derived exosomes transport specific mRNAs such as Il1b, CCL2, CCL7, CCL3, and Pf4, while M2c exosomes are enriched with miR-124-3p and mRNAs such as Ctsb, Fxyd2, Ctsd, and Oaz1. M2d exosomes contain factors including FCER1G, TBK1, and Cathepsins B/S, along with miR-21-5p and miR-155-5p, which contribute to neovascularization and modulation of the tumor microenvironment. Collectively, this dynamic molecular cargo highlights the context-dependent nature of macrophage-derived exosome signaling and their diverse roles in health and disease.

Exosomes are small extracellular vesicles, typically 30 to 150 nanometers in diameter, released through the endosomal pathway [43,44]. They act as mediators of communication by shuttling functional cargos such as proteins, lipids, and genetic materials including microRNAs (miRNAs) from one cell to another [43,45]. Exosomes released from macrophages form an essential class within these vesicles, mirroring the activation status and functional activities of their originating cells [21]. These exosomes not only mirror the phenotypic polarization of macrophages but also actively influence immune responses, inflammation, and tissue remodeling in various pathological conditions including autoimmune diseases (Figure 2) [42,46,47].

M1-polarized macrophages release exosomes enriched in pro-inflammatory mediators. These vesicles commonly contain elevated levels of miRNAs such as miR-155 and miR-21, which amplify inflammatory signaling and promote T cell activation [48,49]. The protein content of M1-derived exosomes contains cytokines such as IL-1β and TNF-α, two key mediators that fuel persistent inflammation and autoimmune responses [50]. When released into inflamed tissue, these exosomes can propagate inflammatory signals, recruit additional immune cells, and exacerbate tissue damage [51].

In contrast, exosomes from M2a macrophages carry molecular signals that promote tissue healing and immunosuppression [52,53]. These exosomes are enriched in miRNAs such as miR-223 and miR-146a [54,55], which dampen inflammatory responses and regulate macrophage reprogramming. Their cytokine cargo commonly carries IL-10 and TGF-β, two pivotal regulators that facilitate the resolution of inflammation while promoting tissue regeneration [56]. These vesicles have shown therapeutic potential in preclinical models by modulating immune responses toward tolerance and regeneration.

Exosomes derived from M2b and M2c macrophages exhibit distinct regulatory profiles, reflecting the specialized roles of their parent cells in immune modulation and tissue remodeling [37,57]. M2b macrophage-derived exosomes may contain a range of anti-inflammatory molecules, consistent with their cytokine profile [58]. Evidence from colitis model mice shows that treatment with M2b macrophage exosomes significantly inhibiting cytokines associated with colitis-related inflammation [37]. Exosomes released from M2c macrophages help reestablish extracellular matrix balance by transferring miR-124-3p and triggering the Smad3 cascade driven by TGF-β [57]. Additionally, exosomes from M2d macrophages (tumor-associated macrophages) are deeply involved in driving cancer cell growth, tissue infiltration, spread to distant sites, and the reconfiguration of tumor metabolism. These exosomes are enriched in vascular growth factors and miRNAs that facilitate neovascularization and tissue remodeling, contributing to the shaping of the tumor microenvironment [59,60,61,62].

Importantly, the composition and function of macrophage-derived exosomes are not static but are dynamically shaped by the surrounding microenvironment [63]. Factors such as cytokine gradients, hypoxia, metabolic stress, and pathogen exposure influence macrophage polarization and, consequently, the quantity, composition, and bioactivity of the exosomes they secrete [63,64,65]. This dynamic interplay underscores the context-dependent nature of exosome signaling and highlights the therapeutic potential of modulating macrophage environments to steer exosome-mediated immune responses [66].

Understanding the distinct cargo and effects of exosomes from various macrophage subsets provides valuable insight into their roles in health and disease. In autoimmune conditions, where dysregulated macrophage activation contributes to persistent inflammation and tissue damage, targeting or harnessing exosome signaling offers a promising strategy for restoring immune balance.

## 4. Macrophage-Derived Exosomes in Autoimmune Diseases

**Figure 3:**
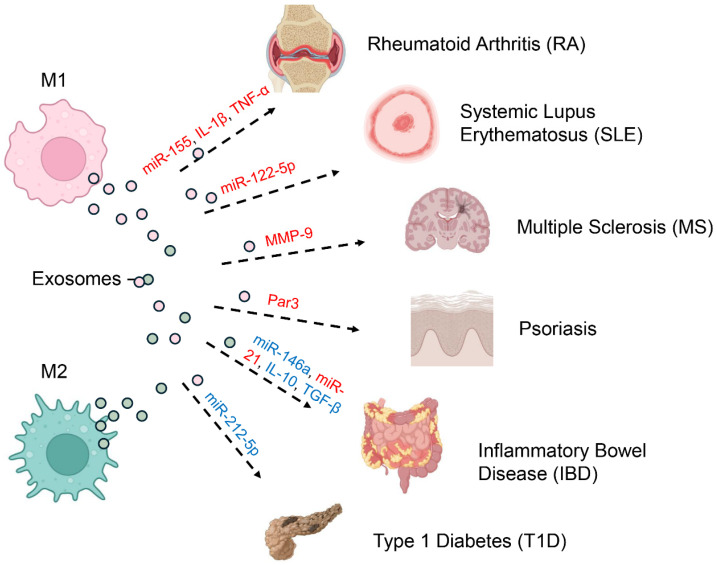
Pathogenic and Protective Roles of Macrophage-Derived Exosomes in Autoimmunity. Macrophage-derived exosomes play dual roles in autoimmunity, either driving pathology or supporting resolution, depending on the polarization state of their parent cells and the cargo they deliver (red indicates pathogenic effects; blue represents protective functions). Exosomes from M1 macrophages, which adopt a pro-inflammatory phenotype, promote disease progression by transporting molecules such as miR-155, IL-1β, and TNF-α in rheumatoid arthritis (RA). In systemic lupus erythematosus (SLE), M1-derived exosomes enriched in miR-122-5p perpetuate systemic autoimmunity, while MMP-9–containing exosomes disrupt the blood–brain barrier in multiple sclerosis (MS), and Par3 exosomes enhance pathogenic signaling in psoriasis. By contrast, M2 macrophage–derived exosomes are often protective. For example, in inflammatory bowel disease (IBD), they deliver anti-inflammatory cargo including miR-146a, miR-21, IL-10, and TGF-β, thereby promoting tissue repair and immune regulation. Nonetheless, M2 exosomes can also have pathogenic roles, as seen in type 1 diabetes (T1D), where miR-212-5p contributes to disease onset.

Immune-mediated disorders arise from misregulated defenses that mistakenly attack self-tissues, leading to chronic inflammation and tissue damage [67,68]. Increasingly, attention has turned to macrophage-derived exosomes as critical modulators of immune responses rather than inert byproducts [69,70]. Depending on their content and the macrophage polarization state, these vesicles can either propagate autoimmune pathology or foster resolution and tissue repair (Figure 3) [22,45].

### 4.1. Rheumatoid Arthritis (RA)

In rheumatoid arthritis, macrophage-derived exosomes are increasingly recognized as amplifiers of chronic joint inflammation [71]. Exosomes released from M1-polarized macrophages within the synovial microenvironment carry inflammatory cargo, notably miRNAs such as miR-155 and cytokines like IL-1β and TNF-α [72,73]. These exosomes can be internalized by resident fibroblast-like synoviocytes (FLS), promoting their activation and proliferation [74]. Activated FLS not only secrete additional pro-inflammatory cytokines but also contribute to cartilage degradation and pannus formation, exacerbating joint destruction [75,76]. In contrast, exosomes derived from M2 macrophages (M2-EVs) have demonstrated potent anti-inflammatory and tissue-protective functions in RA models. M2-EVs can reprogram pathogenic M1 macrophages toward an anti-inflammatory phenotype, reduce synovial inflammation, and limit bone and cartilage damage, with efficacy comparable to methotrexate but without systemic toxicity [77].

### 4.2. Systemic Lupus Erythematosus (SLE)

In SLE, macrophage-derived exosomes participate in the perpetuation of systemic autoimmunity through the transfer of nuclear autoantigens [78,79]. M1-like exosomes encapsulate miRNAs (miR-151a-5p, miR-1180a-5p, miR-1246, and notably miR-122-5p), forming complexes that potently activate plasmacytoid dendritic cells (pDCs) [80,81,82,83]. Upon uptake, these exosomal contents engage endosomal Toll-like receptors (TLR7 and TLR9) in pDCs, triggering the production of large quantities of type I interferons (IFNs) [84,85]. This IFN-driven signature is a hallmark of SLE and sustains the cycle of autoantibody production, immune complex deposition, and systemic inflammation [86]. Emerging data also suggest that exosomes in SLE can alter B cell and T cell function, further entrenching immune dysregulation [87,88,89].

### 4.3. Multiple Sclerosis (MS)

In multiple sclerosis, exosomes released by macrophages promote the disruption of the blood–brain barrier (BBB) and worsen inflammation within the central nervous system (CNS) [90]. Inflammatory exosomes from M1 macrophages contain matrix metalloproteinases (MMPs), including MMP-9, which break down the extracellular matrix and key tight junction proteins essential for maintaining BBB integrity [91,92]. The resulting barrier disruption allows immune cells from the periphery to enter the central nervous system, where they contribute to demyelination and axonal injury [93,94]. Additionally, these exosomes can deliver pro-inflammatory cytokines and miRNAs to resident microglia and astrocytes, perpetuating neuroinflammation and contributing to neuronal injury and clinical progression of MS [95,96].

### 4.4. Inflammatory Bowel Disease (IBD)

Inflammatory bowel diseases are immune-mediated disorders that share a mechanistic overlap with autoimmune diseases, such as dysregulated immune responses and loss of tolerance, but also involve distinct factors like microbiota alterations and epithelial barrier dysfunction [97,98]. Exosomes derived from alternatively activated M2 macrophages, particularly the M2c subtype, exhibit protective and reparative effects in inflammatory bowel disease models [51,99]. These exosomes are loaded with immunosuppressive cytokines, including IL-10 and TGF-β, as well as regulatory microRNAs such as miR-146a and miR-21 [65]. When administered in preclinical models of colitis, M2-derived exosomes reduce epithelial cell apoptosis, enhance mucosal healing, and reinforce epithelial barrier function [37,100]. Their actions collectively diminish local inflammation and promote tissue regeneration, suggesting a potential therapeutic avenue for IBD [101].

### 4.5. Type 1 Diabetes (T1D)

In T1D, islet-resident macrophages play an initiating role in β-cell destruction [102,103]. Recent research indicates that exosomes released by macrophages transport proinflammatory microRNAs, including miR-212-5p, which downregulate SIRT2 and block the SIRT2/Akt/GSK/β-catenin signaling pathway, thereby accelerating the development of autoimmune diabetes [104,105]. These exosomes can modulate both innate and adaptive immune components, fostering an inflammatory milieu within pancreatic islets [102,106,107].

### 4.6. Psoriasis

Psoriasis is characterized by chronic skin inflammation driven by a dysregulated immune response, where macrophage-derived exosomes play a pathogenic role [108]. Macrophage-derived exosomes carrying Par3 can promote the uneven division of basal stem cells and trigger the Par3/mInsc/LGN signaling cascade, thereby influencing the progression of psoriasis [109]. While the pro-inflammatory roles are established, ongoing research is exploring the therapeutic potential of M2 macrophage-derived exosomes to mitigate skin inflammation and restore immune balance [110,111].

## 5. Therapeutic Potential and Challenges

The dual nature of macrophage-derived exosomes as mediators of both pathology and repair has sparked growing interest in their therapeutic potential. With their inherent biocompatibility, ability to traverse biological barriers, and capacity to deliver complex cargo to target cells, exosomes are emerging as attractive candidates for cell-free therapies in autoimmune diseases [45,112,113]. Advances in engineering techniques and macrophage manipulation are now enabling researchers to harness these vesicles for therapeutic purposes, although several key challenges remain.

One promising strategy involves the in vitro polarization of macrophages under defined conditions to produce exosomes with desirable immunomodulatory properties. For example, inducing an M2-like phenotype with interleukin-4 or interleukin-10 can generate exosomes enriched in anti-inflammatory microRNAs and cytokines [114,115,116]. These vesicles have shown the ability to inhibit harmful immune activity and support tissue regeneration in models of autoimmune arthritis, colitis, and neuroinflammation [117]. This approach allows for scalable production and quality control of exosomes suitable for therapeutic applications.

**Figure 4:**
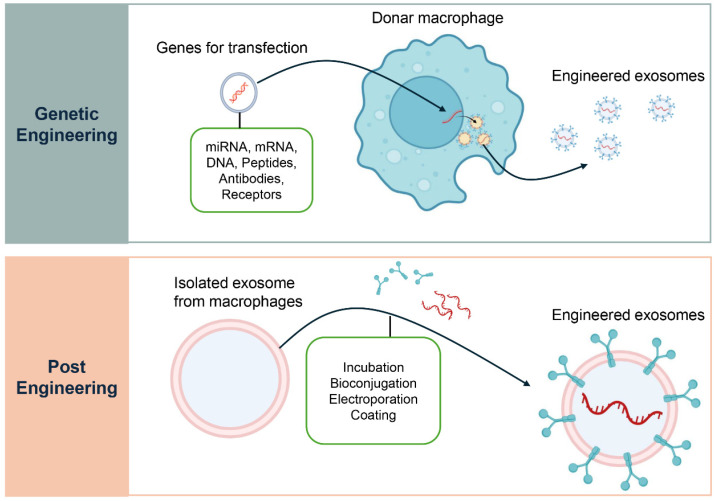
Engineering Strategies for Macrophage-Derived Exosomes. Two main strategies are used to engineer macrophage-derived exosomes for therapeutic applications. The upper panel, Genetic Engineering, illustrates how donor macrophages can be transfected with specific genes to produce exosomes enriched with therapeutic cargo such as miRNAs, mRNAs, DNAs, peptides, antibodies, or receptors. This approach modifies the parent cell to ensure a continuous supply of exosomes with defined properties. The lower panel, Post-Engineering, depicts methods for modifying exosomes after their isolation from macrophages. These include techniques such as incubation, bioconjugation, electroporation, and surface coating, which enable loading of therapeutic agents or functional modifications to enhance targeted delivery to specific cells or tissues.

In addition to their native contents, macrophage-derived exosomes can be modified to transport targeted therapeutic molecules (Figure 4) [118]. Parental cell engineering involves genetically or epigenetically modifying donor macrophages to overexpress therapeutic miRNAs, siRNAs, or fusion proteins (e.g., Lamp2b ligand fusions) [119,120]. Exosomes incorporate these cargos during biogenesis, ensuring physiological packaging and stable loading. This approach is particularly suited for enriching exosomes with immunoregulatory molecules that promote regulatory T cell differentiation, although it requires stable cell manipulation and rigorous quality control [121]. Direct loading methods provide complementary strategies [122]. Electroporation transiently permeabilizes exosome membranes to introduce small RNAs. Mechanical methods such as sonication, extrusion, or freeze–thaw cycles allow incorporation of hydrophobic drugs or small molecules, with validation by NTA, TEM, and protein assays. Chemical transfection systems (lipid- or polymer-based) facilitate nucleic acid or small molecule loading but require stringent purification to avoid contaminants [122,123]. Surface functionalization, including covalent modifications, biotin-streptavidin coupling, or genetic fusions, enables the display of targeting ligands, improving tissue and cell specificity while necessitating in vivo studies to assess biodistribution and immune recognition [124,125].

Routes of administration critically influence therapeutic outcomes. Intravenous injection achieves systemic distribution and CNS targeting, particularly when combined with PEGylation or blood–brain barrier–penetrant ligands [126,127]. Intranasal delivery provides non-invasive CNS access, supporting remyelination and reducing microglial activation [128]. Local administration, such as intra-articular injections, concentrates exosomes in joints to suppress synovitis and cartilage loss [129], while oral or rectal delivery enables mucosal repair in IBD [130]. Topical or intradermal routes are being explored for skin disorders to maximize local effects and minimize systemic exposure [131].

These engineering and functionalization strategies collectively demonstrate the versatility of macrophage-derived exosomes for delivering targeted, immunoregulatory interventions with potentially reduced systemic side effects compared to conventional therapies (Table 2).

Compared with adoptive macrophage therapy, exosome-based strategies offer a distinct, context-dependent risk/benefit profile. Exosomes are cell-free, reducing the risk of uncontrolled engraftment, and can be stored and administered with greater flexibility. They are also generally less immunogenic [132]. By contrast, whole macrophages retain active metabolic and signaling functions that enable dynamic adaptation to local cues but pose risks related to survival, phenotypic drift, and regulatory complexity [133,134]. The choice between exosomes and cells should therefore be guided by disease context, including tissue location (local versus systemic), required duration of action, and acceptable safety margins. For instance, in rheumatoid arthritis (RA), exosomes allow repeatable local dosing with minimal systemic immunosuppression, whereas long-term tissue remodeling may favor macrophage-based therapy if safety can be ensured [135]. In multiple sclerosis (MS), blood–brain barrier-targeted exosomes can deliver neuroprotective cargo without the risks associated with introducing live cells into the CNS [90]. In inflammatory bowel disease (IBD), topical or rectal administration of M2-derived exosomes promotes epithelial repair with lower systemic risk compared with systemic macrophage infusion [136].

Despite these advantages, several challenges remain before macrophage-derived exosomes can be translated into clinical therapies. Stability during storage and circulation is a major limitation, as exosomes are prone to degradation and aggregation [137]. Approaches such as cryopreservation, lyophilization, and encapsulation in protective matrices are being explored to extend shelf life and preserve bioactivity [138].

Targeting specificity also remains a challenge. While macrophage exosomes display some degree of intrinsic homing, off-target distribution and uptake by non-immune cells may limit their precision [139,140,141]. Engineering exosomes with targeting ligands or “self” recognition signals may help improve cellular selectivity and reduce immune clearance [142]. Additionally, rapid uptake by the mononuclear phagocyte system can diminish the therapeutic window [143]. Surface modifications such as PEGylation are being tested to extend circulation time and enhance delivery efficiency [144].

Preclinical studies have demonstrated encouraging results in experimental models of rheumatoid arthritis, multiple sclerosis, and inflammatory bowel disease using macrophage-derived exosomes [71]. Although clinical trials utilizing exosomes from other cell sources are underway, trials specifically involving macrophage-derived exosomes remain in early stages or under development [145,146]. Continued research is needed to refine manufacturing protocols, ensure safety, and validate therapeutic efficacy in human subjects.

## 6. Future Perspectives

As knowledge of macrophage biology and exosome function grows, the opportunity to convert these insights into innovative therapies for autoimmune diseases becomes increasingly attainable. Future directions will likely prioritize improving the targeting precision, therapeutic effectiveness, and clinical safety of macrophage-derived exosome therapies, with an emphasis on personalization and integration with existing treatment approaches.

One promising avenue is the development of personalized exosome therapies tailored to individual immune profiles. Since macrophage polarization varies widely among patients and disease stages [147,148,149], profiling a patient’s macrophage subsets—through blood or tissue biomarkers—could guide the selection or engineering of exosomes with optimal therapeutic effects. For example, patients with predominantly M1-driven inflammation may benefit from exosomes enriched in anti-inflammatory mediators derived from M2-like macrophages. Such personalized approaches could increase therapeutic precision and minimize unintended immune suppression.

Another important direction involves combining exosome therapy with conventional immunosuppressive or biologic treatments. Rather than replacing standard care, exosomes could serve as adjunctive agents that promote immune regulation or tissue repair while allowing for lower doses of traditional drugs [112,118]. This could lower the likelihood of systemic adverse effects and improve the longevity of therapeutic responses [150]. In particular, exosomes could be used to reinforce remission induction or maintain immune homeostasis during tapering phases of immunosuppressive therapy [151].

Advances in nanotechnology and synthetic biology are also paving the way for the creation of synthetic exosome mimetics [152,153,154]. These engineered nanoparticles can be designed to mimic the structure and function of natural exosomes while offering greater control over cargo loading, surface composition, and pharmacokinetics. Synthetic mimetics can be customized to display macrophage-derived signatures, allowing for targeted delivery of immunoregulatory molecules with enhanced stability and reproducibility [155]. Such platforms may overcome some of the scalability and standardization issues associated with natural exosome production.

Ultimately, realizing the full potential of macrophage-derived exosome therapy will require continued progress in several areas, including high-throughput manufacturing, rigorous quality control, targeted delivery strategies, and comprehensive clinical testing. As interdisciplinary collaborations between immunologists, bioengineers, and clinicians advance, the field is poised to redefine the treatment landscape for autoimmune diseases with precision-guided, cell-free interventions.

## 7. Conclusions

Macrophage-derived exosomes represent a rapidly emerging frontier in immunotherapy, uniquely combining immunomodulatory potential, cell-free delivery, and precision targeting. By mirroring the functional state of their parent macrophages and actively modulating recipient cells, they serve dual roles in driving pathology and promoting tissue repair in conditions like rheumatoid arthritis and inflammatory bowel disease. Their therapeutic promise lies in versatility—they can be naturally enriched with regulatory molecules or engineered to carry tailored therapeutic cargo, with advantages including small size, low immunogenicity, and efficient intercellular communication. These features position them as promising candidates for next-generation, personalized therapies, particularly when combined with existing immunosuppressive agents. However, key challenges remain, including standardizing production, enhancing stability and targeting, minimizing off-target effects, and ensuring immune evasion, alongside the need for rigorous clinical validation. Ultimately, macrophage-derived exosomes stand at the intersection of innate immunity, molecular therapy, and regenerative medicine, with the potential to redefine treatment strategies and usher in a new era of targeted, cell-free immunotherapy for autoimmune diseases.

## Figures and Tables

**Table 1:** Macrophage functions beyond M1/M2.

Immune Function	Primary Recipient Cells/Effects	Relevance to Autoimmunity
**Innate immune activation**	Enhance DC maturation and antigen presentation; recruit/activate neutrophils and NK cells	Sustains early inflammatory amplification (RA, SLE, MS)
**Antibody production**	Promote B cell activation/plasmablast differentiation and antibody secretion	Drives autoantibody generation and epitope spreading (SLE, T1D)
**T cell modulation**	Shift Th1/Th17 balance, induce Tregs, or enhance effector T cell responses depending on microenvironment	Controls effector vs regulatory balance in tissues (RA, MS, IBD)
**Tissue repair/tolerance**	Promote epithelial/ECM repair and resolution pathways	Favors mucosal healing and inflammation resolution (IBD, RA remission)

**Table 2:** Engineering and functionalization strategies.

Method	Main Cargo Types	Typical Pros	Typical Cons
**Parental cell engineering**	miRNA, protein, membrane ligands	Physiologic loading; stable cargo	Requires cell line engineering
**Electroporation**	siRNA/miRNA	Simple; good for nucleic acids	Possible RNA aggregation
**Sonication/extrusion/freeze-thaw**	Small molecule drugs, hydrophobic cargos	High loading efficiency for drugs	Can alter EV integrity
**Surface conjugation (click chemistry, Lamp2b fusions)**	Targeting ligands/antibodies	Enhances tropism	May change immune recognition
**Microfluidic loading/shear methods**	Nucleic acids, small molecules	Scalable and reproducible	Technology access and optimization required
